# A Rare Case of Cholecystitis Caused by *Raoultella planticola*

**DOI:** 10.1155/2012/601641

**Published:** 2012-05-29

**Authors:** Isabel Teo, Jonathan Wild, Saikat Ray, David Chadwick

**Affiliations:** ^1^Department of Plastic Surgery and Burns, Nottingham City Hospital, Hucknall Road, Nottingham NG51PB, UK; ^2^Department of General Surgery, Chesterfield Royal Infirmary, Calow, Chesterfield S445BL, UK; ^3^Department of Plastic Surgery, Northern General Hospital, Sheffield S57AU, UK

## Abstract

A 62-year-old female presented with right upper quadrant pain. Clinical examination and ultrasound scan were consistent with gallstones and acute cholecystitis. She received 3 days of intravenous Co-amoxiclav and was discharged with 5-days of oral antibiotics with arrangements to return for an elective cholecystectomy. This was performed 5 months later which revealed an inflamed gallbladder and a localised abscess secondary to gallbladder perforation. Fluid from the gallbladder was taken which cultured *Raoultella planticola*, a gram-negative, nonmotile environmental bacteria (Bagley et al. (1981)). This is the first report of biliary sepsis with a primary infection by *R. planticola*. This patient was treated with a 5-day course of oral Co-amoxiclav and made a full recovery.

## 1. Introduction

*R. planticola* is an aquatic, botanic, and soil organism that does not typically cause invasive infections in humans. There have only been 6 reported cases of serious infection described in humans. We hereby report a case of cholecystitis complicated by *R. planticola.*

## 2. Case Report

A 62-year-old female patient presented to the Accident and Emergency Department with abdominal pain and nausea. She described a 3-week history of worsening right upper quadrant pain but denied any nausea, vomiting, or fevers. Her past medical history included coeliac disease, a hiatus hernia, and irritable bowel syndrome. Her regular medications were Mebeverine, Omeprazole, and Movicol with no known drug allergies. She worked as a cleaner, did not smoke, and drank minimal alcohol. Of note, she had not travelled recently, never had instrumentation of her abdomen, and did not have any recent antimicrobial treatments. Specifically, she denied any intentional or accidental ingestion of soil or aquatic material.

On admission, she was apyrexial with normal observations. Significant examination findings were that of localised tenderness in the right upper abdominal quadrant, consistent with acute cholecystitis. Blood tests revealed raised inflammatory markers with mildly deranged liver function tests. (White Cell Count 24.0 × 10^9^/L, Erythrocyte Sedimentation Rate 98, C-Reactive protein 248, Bilirubin 12, Alkaline Phosphatase 189 U/L, Alanine aminotransferase 58 U/L, Gamma-glutamyl Transferase 141 U/L, Albumin 37 g/L). An ultrasound was performed which reported—“The gallbladder is distended, containing debris and calculi. It is thick walled, tender with some pericholecystic fluid around it. There is no drainable abscess or collections. The ultrasound appearances are consistent with an acute cholecystitis. The common bile duct is not dilated, and there are no dilated intrahepatic ducts. Normal appearances of the liver, spleen, and both kidneys.”

She received 3 days of intravenous Co-amoxiclav, and her symptoms resolved. She was discharged with a 5-day course of oral Co-amoxiclav with arrangements to return for an elective laparoscopic cholecystectomy.

Five months after her initial presentation, she reattended electively for a laparoscopic cholecystectomy. Due to adhesions throughout the epigastrium and right upper quadrant, this was converted to an open procedure. The gallbladder was buried in omentum, and there was a chronic abscess cavity due to a localised perforation of the gallbladder. Fluid from the gallbladder was sent for microbiological examination and a partial cholecystectomy performed.

Postoperatively the patient was systemically well. Her observations were normal with no pyrexia, and she had minimal pain. The surgical team was contacted two days after the operation and informed that viable *Raoultella planticola* had been identified by VITEK 2 biochemical identification system with a very good probability of 99%. This was sensitive to Co-amoxiclav, Ciprofloxacin, Cefuroxime, and Tazocin. She was started on oral Co-amoxiclav following discussion with the microbiology team. She continued to make a good recovery and was discharged after a full 7-day course of Co-amoxiclav.

She was reviewed 3 months after discharge with no new complaints.

## 3. Discussion

*R. planticola* is a gram-negative, nonmotile bacilli primarily considered to be environmental bacteria. *Raoultella* was proposed as a genus in 2001, following the analysis of the genus *Klebsiella* [1]. A comparative analysis of the sequences of the 16S rRNA and rpoB genes were analysed, showing the taxonomic heterogeneity of *Klebsiella *which form three clusters; cluster II organisms were characterised by growth at 10 degrees Celsius and utilisation of L-sorbose as a carbon source, and the name *Raoultella* was created as a genus name for this species of cluster II organisms [2, 3]. Until this time, this organism was known that was being part of the *Klebsiella *genus which was first described in 1981 as *Klebsiella planticola* and later in 1983 as *Klebsiella trevisanii*.

Many *Klebsiella* species are indistinguishable by the conventional methods employed routinely in the clinical microbiology laboratory. There are several recommended additional tests [1, 3–5] to confirm the subspecies of *Klebsiella,* but there is currently no standardized test available [6]. In this patient, *R. Planticola* was identified using the automated identification system VITEK 2 with GN Identification Card (bioMerieux) with a probability of 99%. Whilst this is a high probability, confirmation of identification could be strengthened by additional tests or confirmed by a national *Klebsiella* reference laboratory. This was not carried out firstly because the microbiologists were confident with the 99% probability, and secondly as the patient was clinically stable with a good response to Co-amoxiclav.

*R. planticola *has been reported as clinical isolates in humans in sputum, stools, wounds, and urine [7, 8] but is a rare cause of invasive human infections. To date, there have only been 6 reported cases of serious infection caused by this organism in humans.

The first case report was described by Freney et al. [8] in Lyon, France where a 69-year-old patient with *R. planticola *septicaemia was admitted to an intensive care unit 9 days following a mitral valve replacement [9]. In 1986, Freney et al. [9] described a 57-year-old patient from the same intensive care unit with severe pneumonia following a coronary artery bypass graft [8]. In 2007, Alves et al. [10] in Brazil reported acute pancreatitis and a retroperitoneal abscess in a 45-year-old-man [10]. In 2010, O'Connell et al. [11] described a 30-year-old male in Ireland with soft tissue infection following a crush injury to his thumb in a soiled environment [11]. Also in 2010, Wolcott and Dowd [12] reported a 66-year-old male who developed surgical site infection of the lower limb following open reduction and internal fixation of a tibial fracture [12]. Most recently, Yokota et al. [13] described a 65-year-old gentleman in Japan developing septic shock and cholangitis caused by *R. Planticola *[13].

A common theme is that 4 out of the 7 cases had significant comorbidities—Freney et al. [8]; Freney et al. [9]; Alves et al. [10]; Yokota et al. [13]. Another common theme is that 5 of the 7 had some form of trauma or invasive procedure prior to onset of systemic symptoms—Freney et al. [8]; Freney et al. [9]; O'Connell et al. [11]; Wolcott and Dowd [12]; Yokota et al. [13]. The patient in this report did undergo a cholecystectomy; however, this was 5 months after her episode of cholecystitis; hence, her procedure is unlikely to have played a causative role in her infection. This is summarised in Table 1.

## 4. Conclusion

In conclusion, *R. planticola* is an environmental bacterium that can cause serious infections in humans. From previous reports, we have identified the potential risk factors to include invasive medical procedures, trauma with potential soil contamination, and significant comorbidities. Interestingly, the patient in this report does not have any of these risk factors. The clinical implications of this are uncertain; nonetheless, the correct identification of bacterial species is essential to guide antimicrobial treatment and improve clinical care.

## Figures and Tables

**Table 1:** Reports on clinical infection by *R. planticola *in humans.

Reference	Location	Clinical manifestation	Comorbidities	Invasive procedure/trauma prior to infective episode	Treatment	Outcome
Freney et al*.,* [8]	France	Septicaemia	Bacterial endocarditis	Mitral valve replacement	Cefotaxime and Tobramycin	Full recovery

Freney et al., [9]	France	Septicaemia and pneumonia	Coronary artery disease	Postcoronary artery bypass graft	Ceftriaxone	Full recovery

Alves et al., [10]	Brazil	Acute pancreatitis and retroperitoneal abscess	Pneumonia and alcohol excess	Nil	Imipenem and Amikacin	Full recovery

O'Connell et al., [11]	Ireland	Cellulitis of the thumb	Nil	Crush injury from hammer	Benzyl penicillin, Flucloxacillin, Clindamycin, and Ciprofloxacin	Full recovery

Wolcott and Dowd., [12]	USA	Surgical site infection following *ORIF of the left tibia	Nil	*ORIF	Cephalexin, Clindamycin, and Ertapenem	Full recovery

Yokota et al., [13]	Japan	Cholangitis	Metastatic apocrine adenocarcinoma of the neck	**ERCP	Cefoperazone-sulbactam, Meropenam and piperacillin tazobactam	Clinical improvement

Current report	UK	Cholecystitis	Coeliac disease, hiatus hernia.	Nil	Co-amoxiclav	Full recovery

*ORIF: Open reduction and internal fixation.

**ERCP: Endoscopic retrograde cholangiopancreatography.
